# A Printed Organic Amplification System for Wearable Potentiometric Electrochemical Sensors

**DOI:** 10.1038/s41598-018-22265-1

**Published:** 2018-03-02

**Authors:** Rei Shiwaku, Hiroyuki Matsui, Kuniaki Nagamine, Mayu Uematsu, Taisei Mano, Yuki Maruyama, Ayako Nomura, Kazuhiko Tsuchiya, Kazuma Hayasaka, Yasunori Takeda, Takashi Fukuda, Daisuke Kumaki, Shizuo Tokito

**Affiliations:** 1 Research Center for Organic Electronics (ROEL), Yamagata University, 4-3-16 Jonan, Yonezawa, Yamagata 992-8510 Japan; 2 Functional Polymers Research Laboratory, Tosoh Corporation, 1-8 Kasumi, Yokkaichi, Mie 510-8540 Japan

## Abstract

Electrochemical sensor systems with integrated amplifier circuits play an important role in measuring physiological signals via *in situ* human perspiration analysis. Signal processing circuitry based on organic thin-film transistors (OTFTs) have significant potential in realizing wearable sensor devices due to their superior mechanical flexibility and biocompatibility. Here, we demonstrate a novel potentiometric electrochemical sensing system comprised of a potassium ion (K^+^) sensor and amplifier circuits employing OTFT-based pseudo-CMOS inverters, which have a highly controllable switching voltage and closed-loop gain. The ion concentration sensitivity of the fabricated K^+^ sensor was 34 mV/dec, which was amplified to 160 mV/dec (by a factor of 4.6) with high linearity. The developed system is expected to help further the realization of ultra-thin and flexible wearable sensor devices for healthcare applications.

## Introduction

Wearable sensor systems that enable *in situ* monitoring of biological data from the human body have been expected as promising devices for healthcare and medical purposes^1,2^. Specifically, electrochemical sensors can be utilized to detect a variety of biological markers in bodily fluids such as perspiration, saliva, urine and blood^3,4^. Perspiration from the human body, for example, contains biomarkers such as glucose, lactate, sodium ions, potassium ions, and chloride ions^5^. Glucose and lactate levels in bodily fluids are usually measured using an amperometric measurement method where enzyme-based sensors produce electric current caused by a redox reaction. On the other hand, sodium, potassium, and chloride ions in aqueous solutions are usually measured using a potentiometric measurement method with ion-sensitive membranes. Since the signal levels from potentiometric sensors are typically very low (a few hundreds mV), they should be read by an integrated sensing system equipped with amplifiers having a high input impedance^6,7^. This system plays an important role in *in situ* human perspiration analysis.

Signal processing circuits using organic thin-film transistors (OTFTs) have the potential for realizing extremely thin, lightweight, and flexible wearable sensor devices because they can be processed directly on plastic films possessing a small Young’s modulus and provide a high degree of biocompatibility^2,8,9^. Printability is also a benefit of OTFTs because organic materials can be dissolved in organic solvents, which enables low-cost roll-to-roll manufacture of large-area devices on flexible substrates^10,11^. By utilizing these features, sensor devices that combine OTFTs and physical sensors, such as pressure sensors^12^ and temperature sensors^13^ have been reported, which demonstrated the compatibility and usability of OTFTs for sensor applications. In addition to physical sensors, chemical sensors including pH sensors^14–16^, ion sensors^17–19^, enzymatic sensors^20^, and protein sensors^21,22^ can be implemented with extended-gate OTFT sensors. Organic electrochemical transistors (OECTs) have also been demonstrated.

Few applications of OTFT-based common-source circuits for amplifying small signals from sensors have been reported to date^23–26^. However, without a feedback loop, these amplifiers have drawbacks in low gain controllability, nonlinearity in their amplification characteristics, and difficulty in tuning the input signal to a narrow input-voltage window.

Here, we demonstrate a novel potentiometric electrochemical sensing system employing two OTFT-based negative-feedback inverters. The first inverter connects to the potentiometric sensor electrode amplifies the signal with a tunable gain of 3.1–8.3 and a high linearity. The second inverter connects with the reference electrode and is used for the self-adjustment of the offset voltage. A potassium ion sensor with ion-sensitive membrane was used as a potentiometric sensor. The ion concentration sensitivity of the sensor was 34 mV/dec, which was amplified to 160 mV/dec (by a factor of 4.6) with the developed amplification system. The inverters were fabricated using OTFTs employing a blend of a small molecular p-type semiconductor, 2,7-dihexyl-dithieno[2,3-*d*;2′,3′-*d*′]benzo[1,2-*b*;4,5-*b*′]dithiophene (DTBDT-C_6_), and polystyrene (PS) for the active layer that we have previously reported. A pseudo-CMOS logic design was chosen for the inverters to obtain high gain and rail-to-rail operation, and was adapted to the proposed system because it provided controllability of the switching voltage and closed-loop gain.

## Result and Discussion

### Amplification System Based on Organic Inverter Circuits

**Figure 1:**
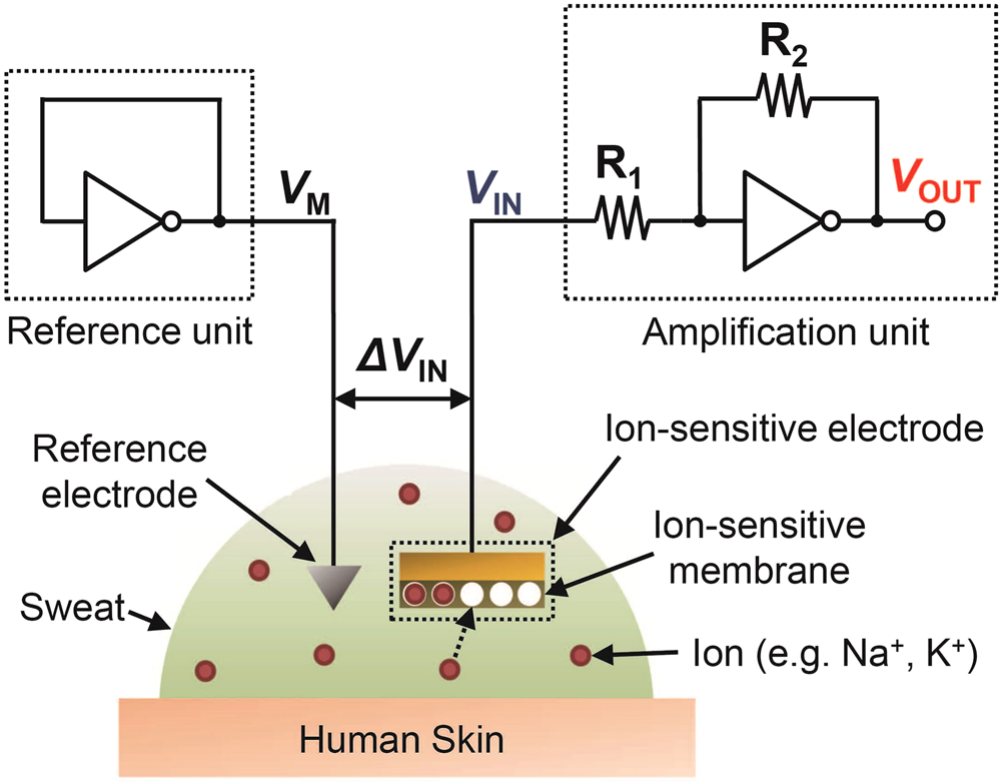
A gain-tunable amplification system for potentiometric sensors. The system is composed of two inverters, two resistors and a potentiometric sensor (e.g. ion sensor).

Figure 1 Shows the configuration of the developed system for potentiometric sensing, which consists of three components: an ion sensor, an amplification unit, and a reference unit. The potassium ion (K^+^) sensor has a membrane on its surface to selectively detect K^+^. The voltage between the ion-sensitive electrode (ISE) and the reference electrode (Ag/AgCl), *E*, varies with the activity (or concentration for a dilute solution) of K^+^, ${a}_{{{\rm{K}}}^{+}}$ (or [K^+^]), according to the Nernst equation: $E={E}_{0}+(\frac{RT}{F}){\rm{l}}{\rm{n}}\,{a}_{{{\rm{K}}}^{+}}$, where *E*_0_ is the standard potential, *R* is the ideal gas constant, *T* is the temperature, and *F* is the Faraday constant. Hence, the sensitivity is usually evaluated by $\frac{dE}{d({\rm{l}}{\rm{o}}{\rm{g}}\,[{{\rm{K}}}^{+}])}$ in units of V/dec. The amplification unit is comprised of an inverter and two resistors, and amplifies the voltage *E* by a predefined gain. Assuming that the open-loop gain of the inverter is sufficiently high, the output voltage of the amplification unit is given by:

1 $${V}_{{\rm{O}}{\rm{U}}{\rm{T}}}={V}_{{\rm{M}}}-\frac{{R}_{2}}{{R}_{1}}({V}_{{\rm{I}}{\rm{N}}}-{V}_{{\rm{M}}})$$

Here, *V*_OUT_ and *V*_IN_ are the output and input voltage of the amplification unit, respectively, and *V*_M_ is the switching voltage of the inverter. In Eq. 1, the input and output voltages have a common offset of *V*_M_, which means that the *V*_IN_ must be close enough to the *V*_M_ for the *V*_OUT_ not to saturate. For this reason, a reference unit was used to set the voltage of the reference electrode at *V*_M_. Finally, the output voltage is given by *V*_OUT_ = *V*_M_−(*R*_2_/*R*_1_) *E*. Either CMOS, PMOS or NMOS inverters can be used for this system as long as they exhibit a high open-loop gain and small variations in *V*_M_. In this work, we employed the PMOS inverters that have a high gain, small *V*_M_ variations, and low operation voltages.

### Fabrication and Characterization of Potassium Ion Sensors

**Figure 2:**
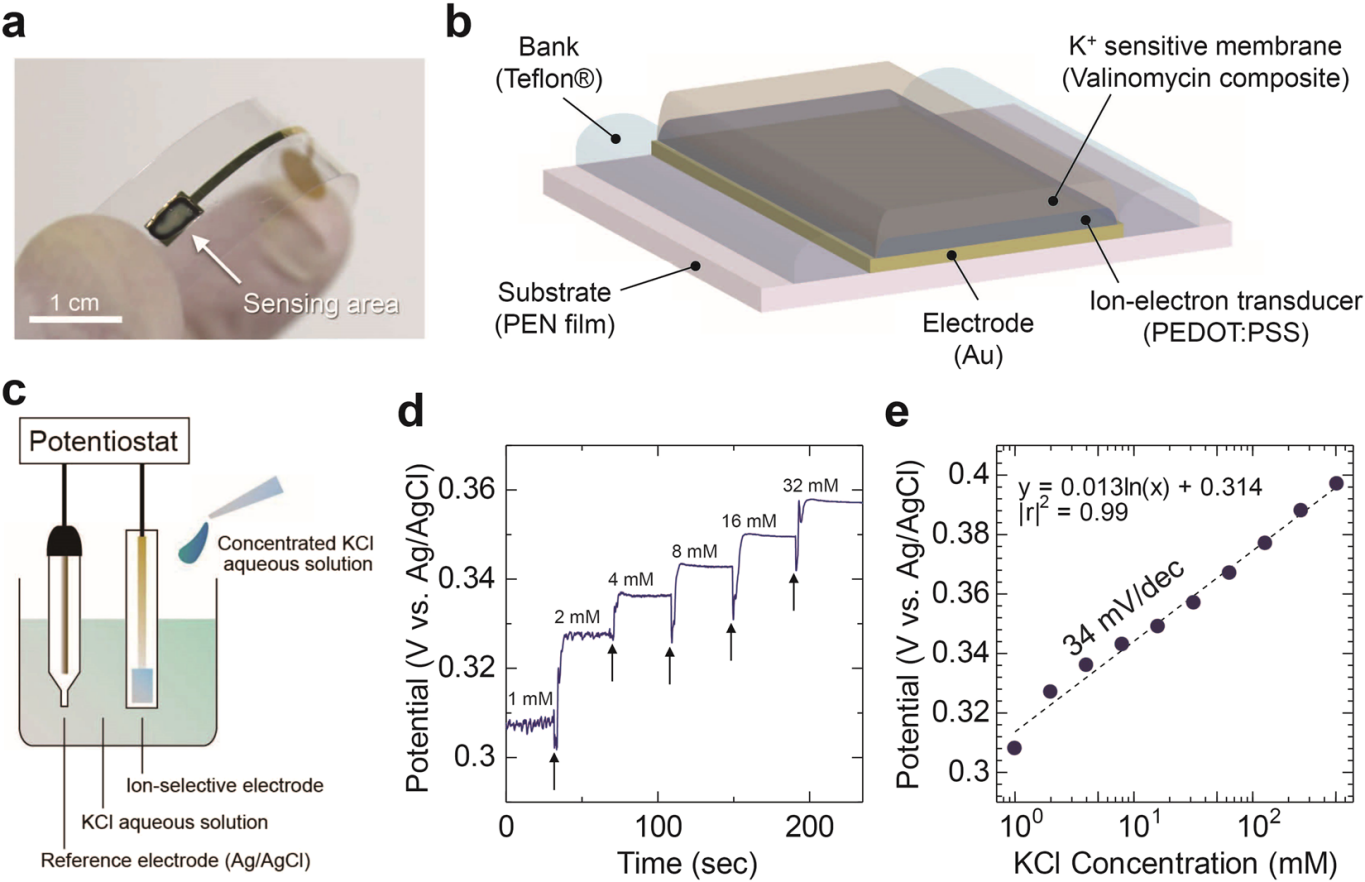
Schematic structure and characteristics of the K^+^ sensor. (**a**) Photograph of the fabricated K^+^ sensitive electrode. The sensing area was 14.3 mm^2^. Schematic diagram of (**b**) K^+^ sensitive electrode and (**c**) potentiometric measurement. (**d**) The open circuit potential responses of the K^+^ sensor in KCl aqueous solution. The inset values are the concentration of K^+^ in the solution. The arrows highlight the times at which concentrated KCl aqueous solutions was added to the solution. (**e**) Potential plots extracted from (**d**) as a function of KCl concentration from 1 to 512 mM.

A photograph and schematic showing the structure of the K^+^-sensitive electrode in the sensor assembly are shown in Fig. 2a,b. After vacuum depositing the gold (Au) electrode, a fluoropolymer bank layer was formed at a periphery of the electrode to define the sensing area. The interconnection area of the Au film was also encapsulated by a fluoropolymer. Poly(3,4-ethylenedioxythiophene):polystyrene sulfonate (PEDOT:PSS) was drop-casted onto the sensing region to increase the effective area of the ion-sensitive membrane/electrode interface and suppress drift of the interfacial potential^27^. The K^+^-sensitive membrane, which was formed on a PEDOT:PSS layer, contains valinomycin as a potassium ionophore, potassium tetrakis(4-chlorophenyl)borate as anion excluder, poly(vinyl chloride) and bis(2-ethylehexyl)sebacate as plasticizers. Valinomycin is known as a K^+^-selective carrier and inherently has a permeability specific to K^+^^28^, enabling the selective detection of K^+^ by the membrane.

The response of the ISE in potentiometric measurements was first tested using a commercial potentiostat (Fig. 2c). When a concentrated KCl aqueous solution was added, the potential of the ISE changed stepwise over several seconds as shown in Fig. 2d. The potential at the respective concentrations was stable at a constant value, which enabled the quantitative measurement of K^+^ levels. Figure 2e shows the potential of the ISE as a function of K^+^ concentration, extracted from Fig. 2d. The total potential change was 90 mV when [K^+^] changed from 1 to 512 mM, which results in the sensitivity of 34 mV/dec. Since the obtained sensitivity was smaller than the theoretical value of 59 mV/dec, further optimization of the fabrication processes for the ISE may be required in the future^3^.

### Structure and Electrical Properties of OTFT Devices

**Figure 3:**
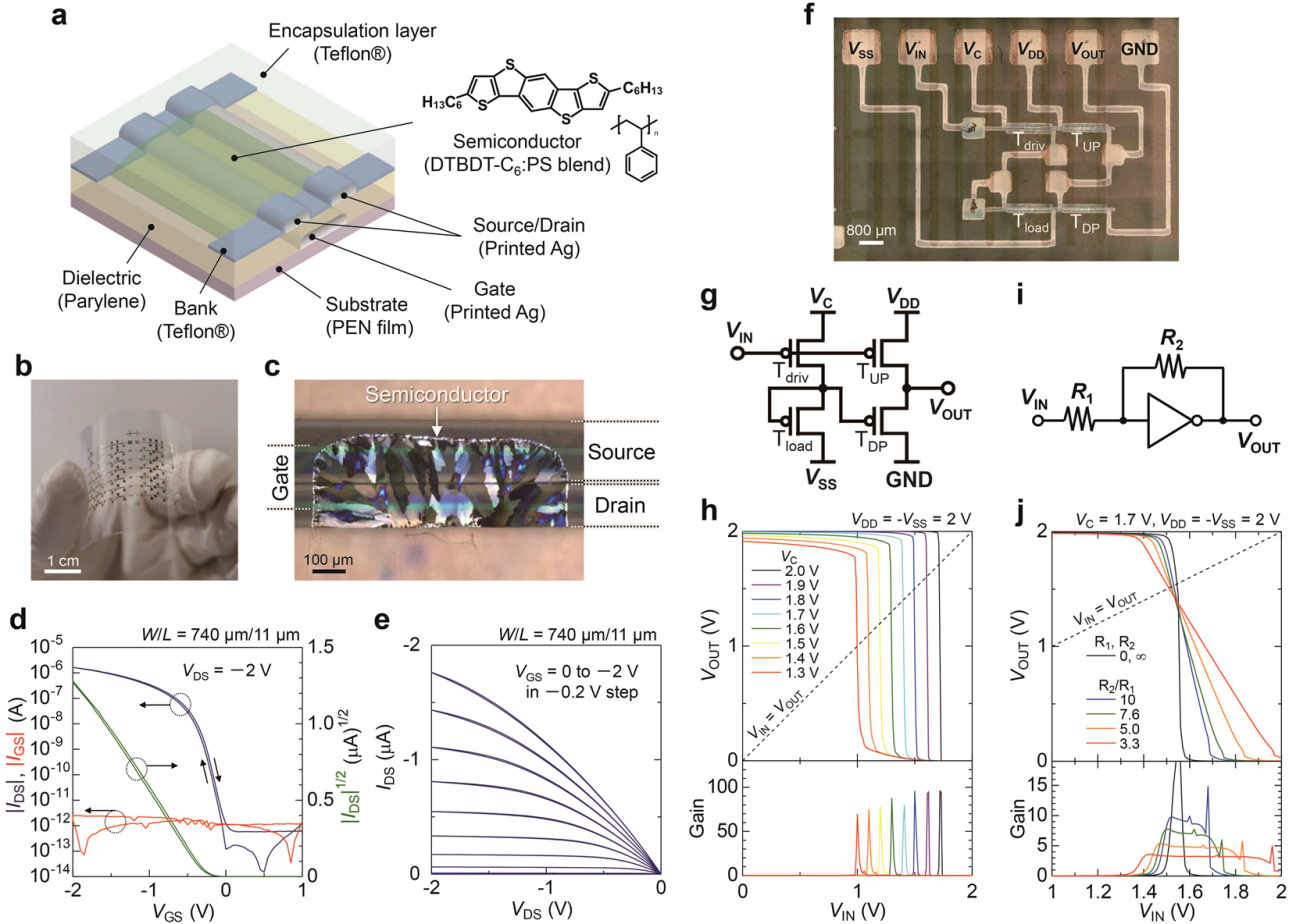
Printed organic semiconductor devices on plastic substrates. (**a**) Schematic structure of the OTFTs and chemical structures of DTBDT-C_6_ and polystyrene (PS). (**b**) Photograph of the devices. (**c**) Cross-polarized optical microscope image of the OTFT channel. (**d**) Transfer curves and (**e**) output curves of the OTFT. (**f**) Optical microscope image and (**g**) circuit diagram of the pseudo-CMOS inverter. (**h**) Static input-output characteristics of the inverter. Output voltage (*V*_OUT_) and small-signal gain (|d*V*_OUT_/d*V*_IN_|) as a function of input voltage (*V*_IN_) at control voltages (*V*_C_) from 2 to 1.3 V in 0.1 V step. (**i**) Circuit diagram of the amplification unit with negative feedback. (**j**) *V*_OUT_ and gain as a function of *V*_IN_ with and without feedback. For the feedback, *R*_2_ is fixed at 1 GΩ, and *R*_1_ is varied from 100 to 300 MΩ.

Figure 3a shows a schematic illustration of the fabricated OTFT devices. All layers except for the gate dielectric were formed using printing processes at process temperatures below 120 °C. The electrodes were fabricated by inkjet printing of silver nanoparticle ink (average particle size is 5 nm). The fluoropolymer bank layer, semiconducting layer, and encapsulation layer were each printed using a dispenser system. A 150-nm-thick parylene gate dielectric layer was then formed by chemical vapor deposition, which exhibited a root-mean-square (RMS) roughness of 2.0 nm. In the same manner as the ion-sensitive electrodes, the OTFT devices could be fabricated on flexible poly(ethylene naphthalate) (PEN) films as shown in Fig. 3b, because of their low processing temperatures. Figure 3c shows a magnified optical image of the OTFT channel. A fluoropolymer bank layer was used to precisely define the channel width and also to control the crystal growth of organic semiconductor, which leads to uniform morphology. By employing the above-mentioned printing processes, the standard deviation of the channel width (*W*) and length (*L*) were ±8 µm and ±2 µm, respectively. A semiconductor blend of 2,7-dihexyl-dithieno[2,3-*d*;2′,3′-*d*′]benzo[1,2-*b*;4,5-*b*′]dithiophene (DTBDT-C_6_)^24,29^ and polystyrene (PS) was chosen as the active layer material to obtain high mobility and uniform electrical performances^30^. As a result of optimizing of the formulation of the semiconductor-blend ink, controlling the crystal growth direction, and annealing of the semiconductor layers, the standard deviation of the onset voltage and threshold voltage were less than 0.03 V^31^.

Figure 3d shows the transfer characteristics of the OTFT in the saturation region. The dielectric capacitance per unit area was 24.3 nF/cm^2^ at 100 Hz (Figure S1). A mobility of 1.1 cm^2^/Vs, threshold voltage of −0.26 V, and subthreshold slope of 100 mV/dec were obtained at a low supply voltage of 2 V. The gate-source leakage current (*I*_GS_) was less than 3 pA. According to the output curve in the linear region shown in Fig. 3e, the semiconductor/electrode contacts exhibited ohmic rather than Schottky behavior. The on-current and threshold voltage were stable under positive and negative gate-bias stress for an hour (Figure S2).

Organic Inverter Circuits with Negative Feedback Inverter circuits used in the amplification system were successfully fabricated by employing the printed OTFTs. Figure 3f,g shows a photograph of the circuit diagram for the fabricated inverter. The inverter design was a pseudo-CMOS design^32^ configured only with p-type OTFTs, enabling high open-loop gain and rail-to-rail input and output signals. The first and second stage are a depletion load inverter^33^ and a level shifter, respectively. Figure 3 h shows the output voltage (*V*_OUT_) and open-loop gain as functions of input voltage (*V*_IN_). The rail-to-rail inverter characteristics were observed, such that *V*_OUT_ switches from *V*_DD_ to zero as *V*_IN_ was swept from zero to *V*_DD_. Rail-to-rail operation is an essential requirement for amplifiers in order to obtain an operating voltage range as large as the supply voltage.

The switching voltage (*V*_M_) was defined as the crossing point of the static input-output characteristics of the inverter and the *V*_IN_ = *V*_OUT_ line. The *V*_M_ value was close to 2 V at supply voltages of *V*_DD_ = *V*_C_ = −*V*_SS_ = 2 V, while a *V*_M_ value near *V*_DD_/2 was more desirable. Precise control of *V*_M_ was enabled by tuning the first-stage supply voltage (*V*_C_). As shown in Figure 3 h, the *V*_M_ value varied linearly with *V*_C_, without significant reduction of the open-loop gain of greater than 70.

The closed-loop gain of the inverter was controlled by a negative feedback using two resistors, as shown in Fig. 3i. Assuming that the input-output characteristics of the inverter in the vicinity of *V*_M_ is expressed as *V*_OUT_ = *V*_M_−*A*_open_(*V*_IN_−*V*_M_), the relation between *V*_OUT_ and *V*_IN_ with the negative feedback is represented by the following equation:

2 $${V}_{{\rm{O}}{\rm{U}}{\rm{T}}}={V}_{{\rm{M}}}-\frac{{R}_{2}}{{R}_{1}+\frac{{R}_{1}+{R}_{2}}{{A}_{{\rm{o}}{\rm{p}}{\rm{e}}{\rm{n}}}}}({V}_{{\rm{I}}{\rm{N}}}-{V}_{{\rm{M}}})$$

(see Figure S3 for details). Consequently, the closed-loop gain is given by:

3 $$Gain=\frac{{R}_{2}}{{R}_{1}+\frac{{R}_{1}+{R}_{2}}{{A}_{{\rm{o}}{\rm{p}}{\rm{e}}{\rm{n}}}}}$$

where *A*_open_ is the open-loop gain of the inverter. In the case of an adequately high open-loop gain, *A*_open_ ≫ (*R*_1_ + *R*_2_)/*R*_1_, the closed-loop gain is given by *R*_2_/*R*_1_. These formula indicate that the closed-loop gain can be simply controlled by the two resistors if the open-loop gain is sufficiently high. Figure 3j shows *V*_OUT_ and the closed-loop gain as a function of the *V*_IN_. The closed-loop gain could be controlled from 3.1 to 8.6 by changing the resistance ratio (*R*_2_/*R*_1_) from 3.3 to 10 (*R*_2_ is kept at 1 GΩ). Thus, high controllability of the closed-loop gain was obtained as a result of negative feedback and the high open-loop gain of the inverter (*A*_open_ > 70). Figure S4 shows the dependence of the closed-loop gain on the ratio of *R*_2_/*R*_1_. Although the experimental closed-loop gain deviated from *R*_2_/*R*_1_ by up to 14%, it fit well with the Eq. 3. This deviation can be reduced further by increasing the open-loop gain of the inverters. Figure 3j also indicates that the closed-loop gain at each *R*_2_/*R*_1_ value is nearly constant over a wide *V*_IN_ range, unless *V*_OUT_ is saturated, which means the fabricated amplifiers possessed high linearity.

Here, we compare the above characteristics with those of the common-source amplifiers without feedback. The gain of a resistor-load common-source amplifiers, for example, is given by $-R\mu {C}_{{\rm{i}}}\frac{W}{L}({V}_{{\rm{I}}{\rm{N}}}-{V}_{{\rm{T}}{\rm{H}}})$, which includes several parameters, such as the load resistance *R*, the mobility *μ*, the capacitance per unit area *C*_i_, the channel width *W*, the channel length *L*, and the threshold voltage *V*_TH_. Therefore, each of the parameters must be precisely controlled to reproducibly set the resulting gain. By contrast, the gain of the present feedback system depends only on the two resistances, *R*_1_ and *R*_2_, enabling the high degree of gain control. Another disadvantage of feedback-less amplifiers is that their gain depends on the input voltage *V*_IN_, which means that the amplifiers are inherently nonlinear. Based on these considerations, pseudo-CMOS inverters with negative feedback are suitable for the amplification of quantitative measurements because of the high degree of gain controllability and high linearity.

### Application of Organic Inverter-based Amplification System to Ion Sensors

**Figure 4:**
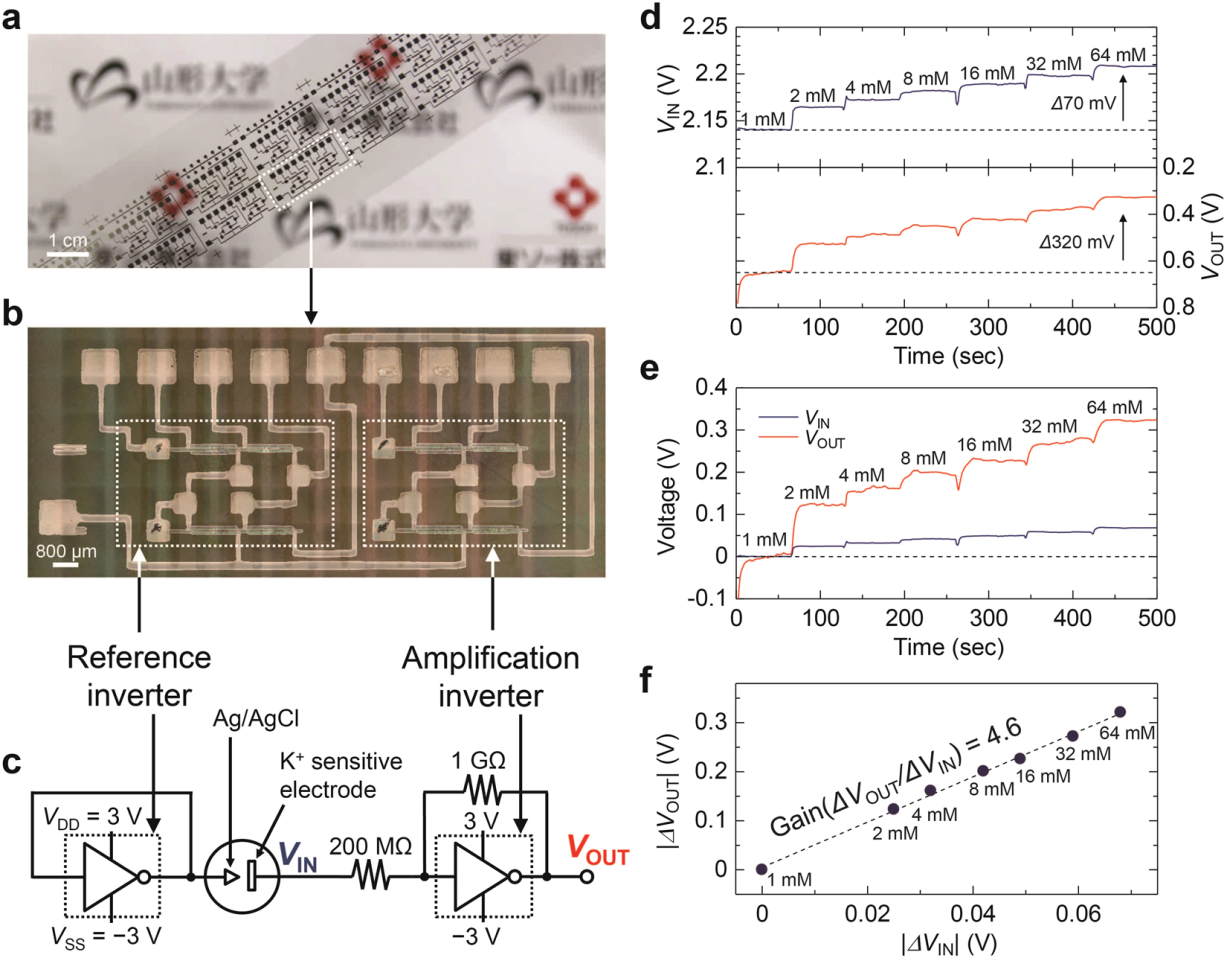
Amplification of small signals from a K^+^ sensor using the printed organic circuits. (**a**) Photograph and (**b**) optical microscope image of the reference and amplification units. (**c**) Entire system for K^+^ sensing. Supply voltage (*V*_DD_ = −*V*_SS_) and control voltage (*V*_C_) of both reference inverter and amplification inverter was set to 3 V and 2 V, respectively. (**d**) Raw and (**e**) zero-adjusted input (*V*_IN_) and output voltage (*V*_OUT_) of the amplifier system. Concentration of KCl aqueous solution was from 1 to 64 mM. (**f**) Absolute values of *V*_OUT_ change (|*ΔV*_OUT_|) vs. those of *V*_IN_ change (|*ΔV*_IN_|) extracted from (**e**). The slope from least squares fitting (*ΔV*_OUT_/*ΔV*_IN_) corresponds to the amplification factor of the amplifier.

The potassium ion (K^+^) sensing system was fabricated with printed organic pseudo-CMOS inverters, as shown in Fig. 4a,b. According to the amplification characteristics shown in Fig. 3j, the electrical potential of the K^+^-sensitive electrode has to be tuned to a point close to *V*_M_. Therefore, the reference electrode potential should be set to *V*_M_. To generate the voltage *V*_M_, the input and output terminals of the reference inverter were connected to each other, as shown in Fig. 4c.

Here, a key requirement is that the difference in *V*_M_ between the inverter for the reference unit and that for the amplification unit is as small as possible, because a uniform and reproducible *V*_M_ results in a reduction of the offset level and the realization of a high amplification factor. However, a major issue in solution-processed or printed OTFTs is the relatively large variations in their electrical properties^10,11^. Therefore, in the fabrication of an amplification system based on printed OTFTs, reductions in these variations should be one of the more important requirements. In this work, the maximum difference in *V*_M_ was 150 mV, as enabled by our previous work^31^, which optimized the formation process of the blended DTBDT-C_6_:PS active layer. One inverter pair was randomly chosen from those fabricated and was used for the amplification system. The difference in *V*_M_ for the chosen inverter pair was 40 mV (Figure S5), indicating acceptable uniformity for its application to analog circuits. In order to insure uniformity of the printed OTFT devices, we continue to study ways to reduce variations in its electrical characteristics.

Figure 4c shows the circuit diagram of the entire amplification system. The supply voltages for both the reference inverter and amplification inverter were set to *V*_DD_ = −*V*_SS_ = 3 V and *V*_C_ = 2 V. The *R*_2_/*R*_1_ was 1 GΩ/200 MΩ = 5. The K^+^ concentration was increased stepwise from 1 mM to 64 mM, and the input voltage (*V*_IN_) and output voltage (*V*_OUT_) of the amplification unit were measured simultaneously (Fig. 4d). The *V*_IN_ exhibited the sensitivity of 39 mV/dec to the K^+^ concentration, which is consistent with the sensitivity in Fig. 2e. The total changes in *V*_IN_ and *V*_OUT_ were 70 mV and 320 mV, respectively. Figure 4e shows the shifts in *V*_IN_ and *V*_OUT_ relative to both voltages at 1 mM, showing that the system undoubtedly functioned as an amplifier. Figure 4f shows the change of *V*_OUT_ as a function of the change of *V*_IN_ extracted from Fig. 4e. The slope of the line (d*V*_OUT_/d*V*_IN_) was estimated to be 4.6, corresponding to the predefined amplification factor, a value that was very consistent with the gain in Fig. 3j. Thus, an amplification system based on printed organic inverter circuits successfully demonstrated their potential application to the potentiometric electrochemical sensors. We note that, without the presence of the PEDOT:PSS layer in the ISE, non-negligible potential drifts were observed using the present amplification system (see Figures S6 and S7). This is because the current of $\frac{{V}_{{\rm{I}}{\rm{N}}}-{V}_{{\rm{O}}{\rm{U}}{\rm{T}}}}{{R}_{1}+{R}_{2}}$ flowed at the ISE and changed the distribution of ions near the electrode. The PEDOT:PSS layer helps to reduce the potential drift by increasing the effective area of the ion-sensitive membrane/electrode interface^27^.

In summary, a novel potentiometric electrochemical sensing system based on organic thin-film transistors was developed for realizing ultra-thin and flexible wearable device applications. The K^+^ sensitive electrode exhibited the distinct changes of the potential depending on the concentration of K^+^ in aqueous solution, which enabled the quantification of K^+^ concentration levels. Inverters using a pseudo-CMOS logic configuration were formed using TFT devices based on a DTBDT-C_6_:PS blend whose fabrication processes were optimized. The pseudo-CMOS inverters exhibited high open-loop gain (>70), rail-to-rail operation, and uniform switching voltage. Negative feedback was adapted to the amplification system owing to the high controllability of the closed-loop gain and a high linearity. The sensitivity of the K^+^ sensor was amplified by the developed sensing system from 34 mV/dec to 160 mV/dec (a factor of 4.6). These results show the potential for realizing potentiometric electrochemical sensor devices based on printed organic circuits. By combining the advanced features of organic electronics and biosensors, a smart wearable biosensor device, which is flexible, lightweight and low cost, as well as highly sensitive, can be realized for potential healthcare applications.

## Methods

### Fabrication of the Potassium Ion Sensors

2 mg of valinomycin (Wako Pure Chemical Industries), 0.5 mg of potassium tetrakis(4-chlorophynyl)borate (Sigma-Aldrich), 32.7 mg of poly(vinyl chloride) (Sigma-Aldrich), 64.7 mg of bis(2-ethylehexyl)sebacate (Tokyo Chemical Industry) were dissolved in 350 µL of tetrahydrofuran. The ion-sensitive solutions were sealed and stored at 4 °C. 125-µm-thick polyethylene naphthalate (PEN) films (Teonex, Teijin) were used as substrates without cleaning process. A 50-nm-thick Au layer was deposited by thermal evaporation. In order to define the sensing area, a fluoropolymer (5 wt%, Teflon AF1600, DuPont) in Fluorinert (FC-43, 3 M) bank layer was formed onto the substrate except the sensing area, followed by an annealing process of 60 °C for 15 min. in an air ambient. 2 µL of PEDOT:PSS (Clevious P Jet700, Heraeus) as the ion-electron transducer to minimize the potential drift of the sensors was drop-casted onto the area defined by the bank layer, followed by an annealing process of 120 °C for 15 min. in an air ambient. Next, 10 µL of the potassium ion-sensitive solution was drop-casted onto the PEDOT:PSS film, followed by a drying process of 30 °C in an air ambient overnight.

### Fabrication of the Organic Semiconductor Devices

125-µm-thick polyethylene naphthalate (PEN) films (Teonex, Teijin) were used as substrates without cleaning process. A silver nanoparticle ink (average particle size is 5 nm) in hydrocarbon-based solution (NPS-JL, Harima Chemicals) was printed as gate electrodes using an inkjet printer (Dimatix DMP2831, Fujifilm) with 10 pL nozzles. During the inkjet printing process, the substrates and cartridge were kept at 50 and 35 °C, respectively. The substrates were then heated at 120 °C for 30 min. in an air ambient to sinter the silver nanoparticles. The RMS roughness of the resulting electrode was 6.3 nm. A 150-nm-thick parylene (KISCO, diX-SR) gate dielectric layer was then formed by chemical vapor deposition. The RMS roughness of the resulting parylene film was 2.0 nm. Source and drain electrodes were subsequently printed and sintered in the same manner as the gate electrodes. Fluoropolymer (1 wt%, Teflon AF1600, DuPont) in Fluorinert (FC-43, 3 M) bank layers (200 nm thick) were then printed using a dispenser system (Image Master 350 PC, MUSASHI Engineering) at a pattering speed of 20 mm s^−1^ and with a discharge pressure of 6 kPa. During the dispensing process, the plate and nozzle temperatures were kept at 60 and 30 °C, respectively. To apply a self-assembled monolayer (SAM) treatment to source and drain electrodes, the substrates were immersed in a 3 × 10^−2^ mol/L 2-propanol solution of pentafluorobenzenethiol (Tokyo Chemical Industry) for 5 min. at room temperature and rinsed with pure 2-propanol. The SAM treatment changed the work function of the printed silver electrodes from 4.7 to 5.4 eV, which reduces the contact resistance. A solution of DTBDT-C_6_ (0.9 wt%, Tosoh) and polystyrene (0.3 wt%, *M*_W_ ≈ 280,000, Sigma-Aldrich) in toluene was then printed onto the area defined by the bank layer by the dispenser system at a patterning speed of 20 mm s^−1^ and discharge pressure of 1 kPa, while keeping the stage and nozzle temperatures at 30 °C, followed by an anneal at 100 °C in an air ambient for 15 min. to remove the solvent. Finally, an encapsulation layer of Teflon was printed by the dispenser system at 30 °C, with a pattering speed of 8 mm s^−1^ with a discharge pressure of 6 kPa. The substrates were stored at room temperature in an air ambient for three hours to remove the solvent.

### Characterization of the Potassium Ion sensor

The K^+^ sensors were dipped in 10 mM KCl aqueous solution for 1 hour before potentiometric measurements. The potentiometric measurements were carried out using an electrochemical analyzer (ALS612E, BAS). During the measurements, the subject solution was stirred at 400 rpm.

### Characterization of the Organic Semiconductor Devices

The capacitance of the dielectric and CV characteristics of the OTFTs were measured using an LCR meter (ZM2376, NF). The electrical characteristics of the OTFTs and inverter circuits were measured using a semiconductor parameter analyzer (4200A-SCS, Keithley). All electrical measurements were carried out in an air ambient. Optical microscope images of the devices were obtained using a digital microscope (VHX-5000, Keyence).

### Amplification of Small Signals from the Potassium Ion Sensor Using the Organic Semiconductor Devices

Voltage supply and measurements were carried out using a semiconductor parameter analyzer (4200A-SCS, Keithley). The inverters were connected using commercial resistors, a reference electrode, and the K^+^ sensor via coaxial cables. The amplification system was biased (*V*_DD_ = −*V*_SS_ = 3 V, *V*_C_ = 2 V) for 5 min. before the measurements to stabilize their operation.

## Electronic supplementary material

Supplementary Information

## References

[1] Kim D-H (2011). Epidermal electronics. Science.

[2] Kaltenbrunner M (2013). T. An ultra-lightweight design for imperceptible plastic electronics. Nature.

[3] Gao W (2016). Fully integrated wearable sensor arrays for multiplexed *in situ* perspiration analysis. Nature.

[4] Lee H (2017). Wearable/disposable sweat-based glucose monitoring device with multistage transdermal drug delivery module. Sci. Adv..

[5] Sonner Z (2015). The microfluidics of the eccrine sweat gland, including biomarker partitioning, transport, and biosensing implications. Biomicrofluidics.

[6] Yokota T (2012). Sheet-type flexible organic active matrix amplifier system using pseudo-CMOS circuits with floating gate structure. IEEE. Trans. Electron. Devices.

[7] Sekitani T (2016). Ultraflexible organic amplifier with biocompatible gel electrodes. Nat. Commun..

[8] Sekitani T, Zschieschang U, Klauk H, Someya T (2010). Flexible organic transistors and circuits with extreme bending stability. Nat. Mater..

[9] Kuribara K (2011). Organic transistors with high thermal stability for medical applications. Nat. Commun..

[10] Fukuda K (2014). Fully-printed high-performance organic thin-film transistors and circuitry on one-micron-thick polymer films. Nat. Commun..

[11] Pierre A (2014). All-printed flexible organic transistors enabled by surface tension-guided blade coating. Adv. Mater..

[12] Someya T (2004). A large-area, flexible pressure sensor matrix with organic field-effect transistors for artificial skin applications. Proc. Natl. Acad. Sci. USA.

[13] Yokota T (2015). Ultraflexible, large-area, physiological temperature sensors for multipoint measurements. Proc. Natl. Acad. Sci. USA.

[14] Spijkman M-J (2014). Dual-gate organic field-effect transistors as potentiometric sensors in aqueous solution. Adv. Funct. Mater..

[15] Seo, J. *et al.* Broadband pH-sensing organic transistors with polymeric sensing layers featuring liquid crystal microdomains encapsulated by di-block copolymer chains. *ACS. Appl. Mater. Interfaces*. **8**, 23862 (2016). 10.1021/acsami.6b08257 27557404

[16] Scheiblin G, Coppard R, Owens RM, Mailley P, Malliaras GG (2016). Referenceless pH sensor using organic electrochemical transistors. Adv. Mater. Technol..

[17] Scarpa G, Idzko A-L, Yadav A, Thalhammer S (2010). Organic ISFET based on poly (3-hexylthiophene). Sensors.

[18] Schmoltner K, Kofler J, Klug A, List-kratochvil EJW (2013). Electrolyte-gated organic field-effect transistor for selective reversible ion detection. Adv. Mater..

[19] Minami T (2015). S. A mercury (II) ion sensor device based on an organic field effect transistor with an extended-gate modified by dipicolylamine. Chem. Commun..

[20] Tang H, Yan F, Lin P, Xu J, Chan HLW (2011). Highly sensitive glucose biosensors based on organic electrochemical transistors using platinum gate electrodes modified with enzyme and nanomaterials. Adv. Funct. Mater..

[21] Palazzo G (2015). Detection beyond debye’s length with an electrolyte-gated organic field-effect transistor. Adv. Mater..

[22] Minamiki T (2014). Accurate and reproducible detection of proteins in water using an extended-gate type organic transistor biosensor. Appl. Phys. Lett..

[23] Tang W (2016). Low-voltage pH sensor tag based on all solution processed organic field-effect transistor. IEEE Electron Device Lett..

[24] Fukuda K (2015). Printed organic transistors with uniform electrical performance and their application to amplifiers in biosensors. Adv. Electron. Mater..

[25] Svensson P-O, Nilsson D, Forchheimer R, Berggren M (2008). A sensor circuit using reference-based conductance switching in organic electrochemical transistors. Appl. Phys. Lett..

[26] Ji X (2016). Highly sensitive metaboloite biosensor based on organic electrochemical transistor integrated with microfluidic channel and poly(N-vinyl-2-pyrrolidone)-capped platinum nanoparticles. Adv. Mater. Technol..

[27] Bobacka J (1999). Potential stability of all-solid-state ion-selective electrodes using conducting polymers as ion-to-electron transducers. Anal. Chem..

[28] Bhattacharyya P, Epstein W, Silver S (1971). Valinomycin-induced uptake of potassium in membrane vesicles from escherichia coli. Proc. Natl. Acad. Sci. USA.

[29] Gao P (2009). Adv. Mater..

[30] Shiwaku R (2016). Printed 2 V-operating organic inverter arrays employing a small-molecule/polymer blend. Sci. Rep..

[31] Shiwaku R (2017). Printed organic inverter circuits with ultralow operating voltages. Adv. Electron. Mater..

[32] Huang T-C (2011). Pseudo-CMOS: a design style for low-cost and robust flexible electronics. IEEE Trans. Electron Devices.

[33] Nausieda I (2010). Dual threshold voltage organic thin-film transistor technology. IEEE Trans. Electron Devices.

