# Supplementary material for: A Printed Organic Amplification System for Wearable Potentiometric Electrochemical Sensors
Source: Sci Rep. 2018 Mar 2;8:3922. doi: 10.1038/s41598-018-22265-1 (PMC5834464; doi:10.1038/s41598-018-22265-1)
Supplement: Supplementary file 1 — Supplementary Information [file 41598_2018_22265_MOESM1_ESM.pdf]

Supplementary Information

A Printed Organic Amplification System for Wearable Potentiometric Electrochemical Sensors

Rei Shiwaku[1], Hiroyuki Matsui[1,a], Kuniaki Nagamine[1,a], Mayu Uematsu[1], Taisei Mano[1], Yuki Maruyama[1], Ayako Nomura[1], Kazuhiko Tsuchiya[1], Kazuma Hayasaka[1], Yasunori Takeda[1], Takashi Fukuda[2], Daisuke Kumaki[1], and Shizuo Tokito[1,a]

[1]Research Center for Organic Electronics (ROEL), Yamagata University
4-3-16 Jonan, Yonezawa, Yamagata, 992-8510, Japan

[2]Functional Polymers Research Laboratory, Tosoh Corporation
1-8 Kasumi, Yokkaichi, Mie, 510-8540, Japan

[a]Author to whom correspondence should be addressed: h-matsui@yz.yamagata-u.ac.jp (H. Matsui), nagamine@yz.yamagata-u.ac.jp (K. Nagamine), tokito@yz.yamagata-u.ac.jp (S. Tokito)

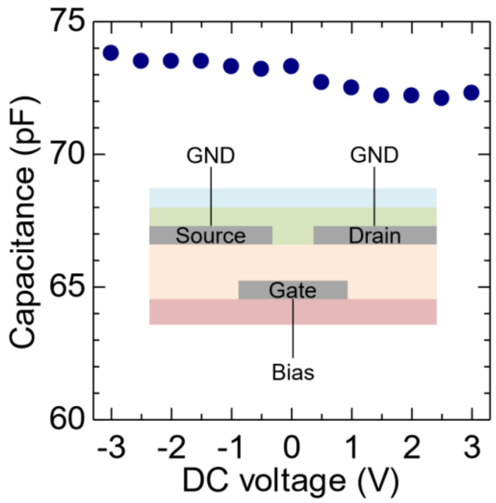

Figure S1. CV characteristics of the DTBDT-C_6:PS blend-based TFTs.

Each plot was measured at a frequency of 100 Hz. Gate overlap area was 0.3 mm^2. The channel width (*W*) and length (*L*) were 740 µm and 11 µm, respectively.

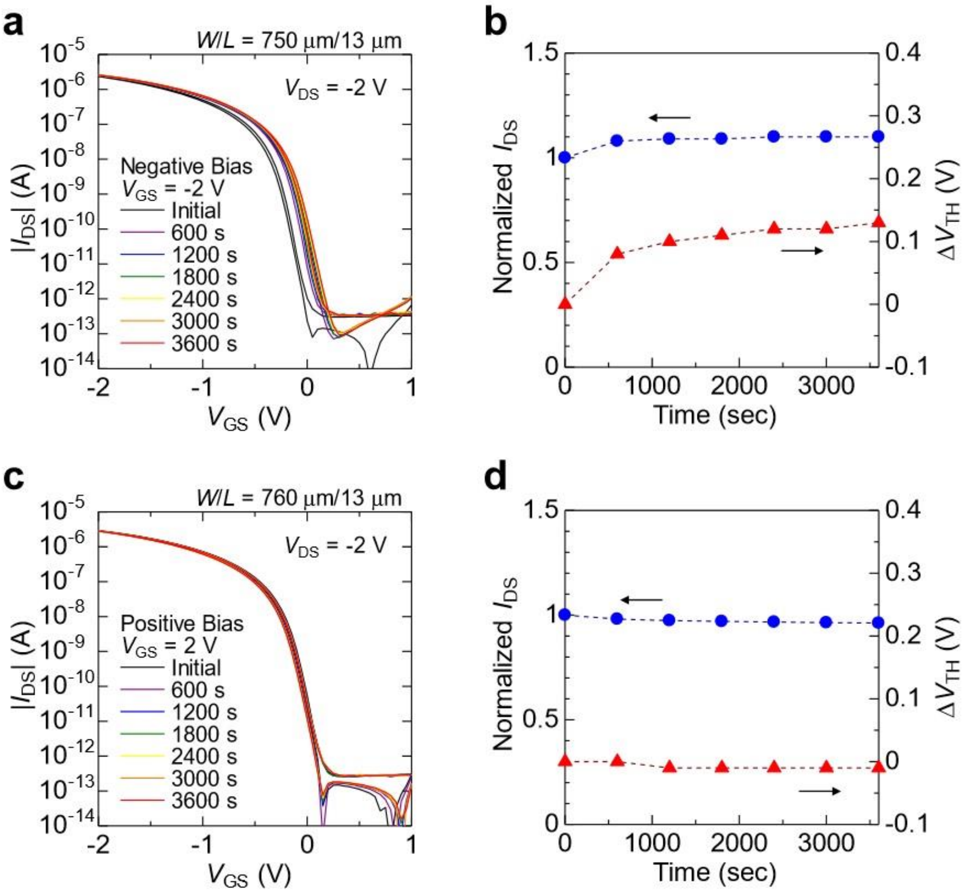

Figure S2. DTBDT-C$_6$:PS blend TFT stability under gate-bias stress.

Transfer curves under (a) negative and (c) positive gate-bias stress. Normalized drain-source current and threshold voltage shift under (b) negative and (d) positive gate-bias stress.

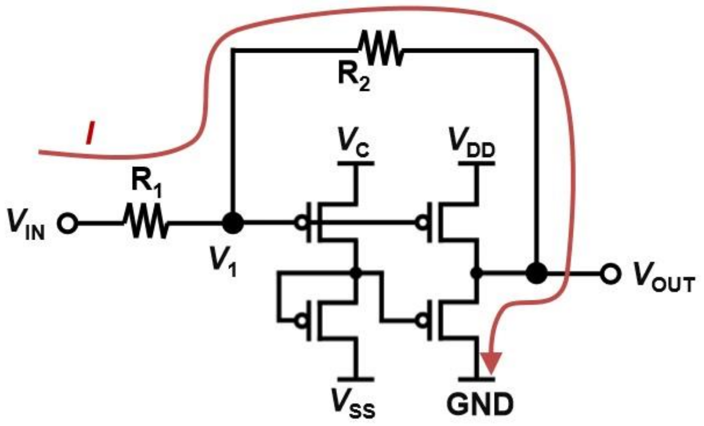

Figure S3. Amplification unit circuit diagram using a pseudo-CMOS logic design.
A single path for current flow (I). Here, the relation between V_{OUT} and V_{IN} is shown below. Taking the voltage drops at each of the section into consideration, following equations can be obtained:

$$V_{OUT} - V_M = -A_{open}(V_1 - V_M), \text{ (S1)}$$

$$V_{IN} - V_1 = R_1 I, \text{ (S2)}$$

$$V_1 - V_{OUT} = R_2 I, \text{ (S3)}$$

where V_M and A_{open} is the switching voltage and the open-loop gain of the inverter, respectively. Removing V_1 and I, V_{OUT} is finally expressed as:

$$V_{OUT} = V_M - \frac{R_2}{R_1 + \frac{R_1 + R_2}{A_{open}}}(V_{IN} - V_M), \text{ (S4)}$$

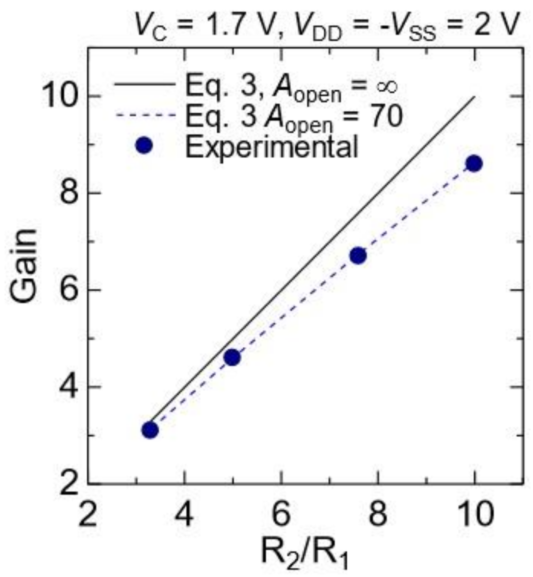

Figure S4. Calculated vs. experimental gain comparisons.

Gain calculated from Eq. 3 and from experiment measurements, as a function of the resistance ratio.

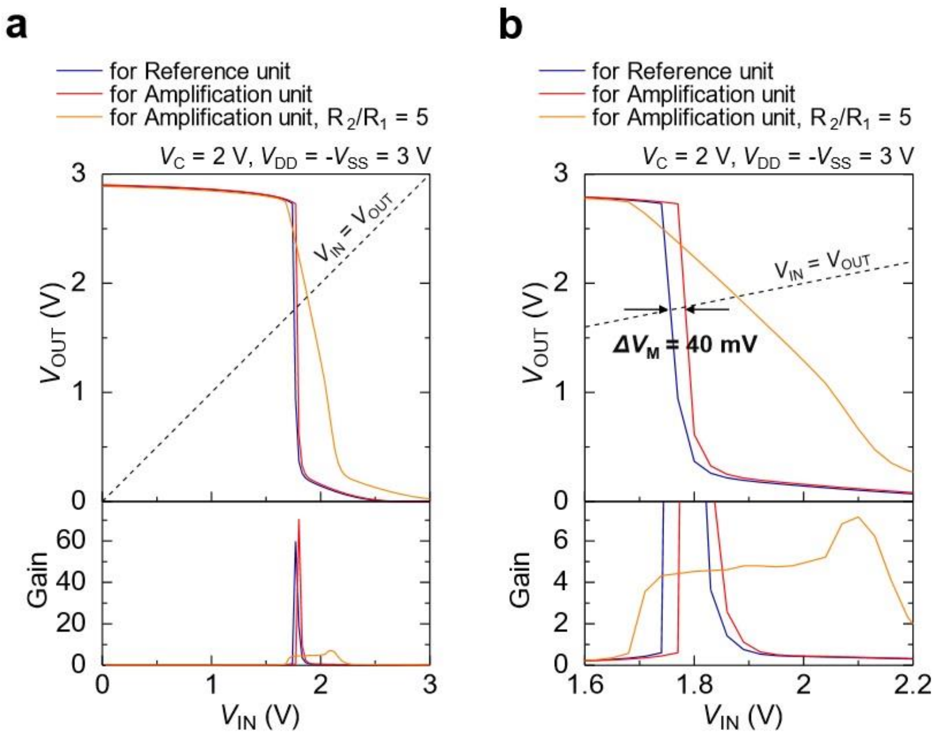

Figure S5. Input-output characteristics of the inverter pair that was used for the K^+ sensing system.

(a) V_{OUT} and gain as a function of V_{IN}. (b) Magnified characteristics of (a). The difference between the reference and amplification inverters was 40 mV, which was less than the K^+ sensor signal of 70 mV.

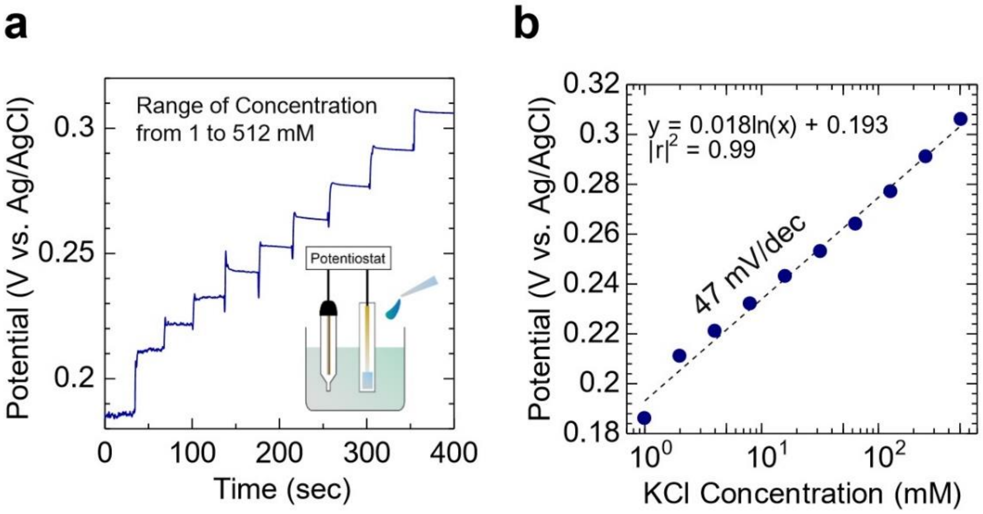

Figure S6. Potentiometric curve of K^+ sensor without a PEDOT:PSS ion-electron transducer. (a) The open circuit potential responses of K^+ sensor without a PEDOT:PSS layer. (b) Potential plots extracted from (a) as a function of KCl concentration (1 to 512 mM).

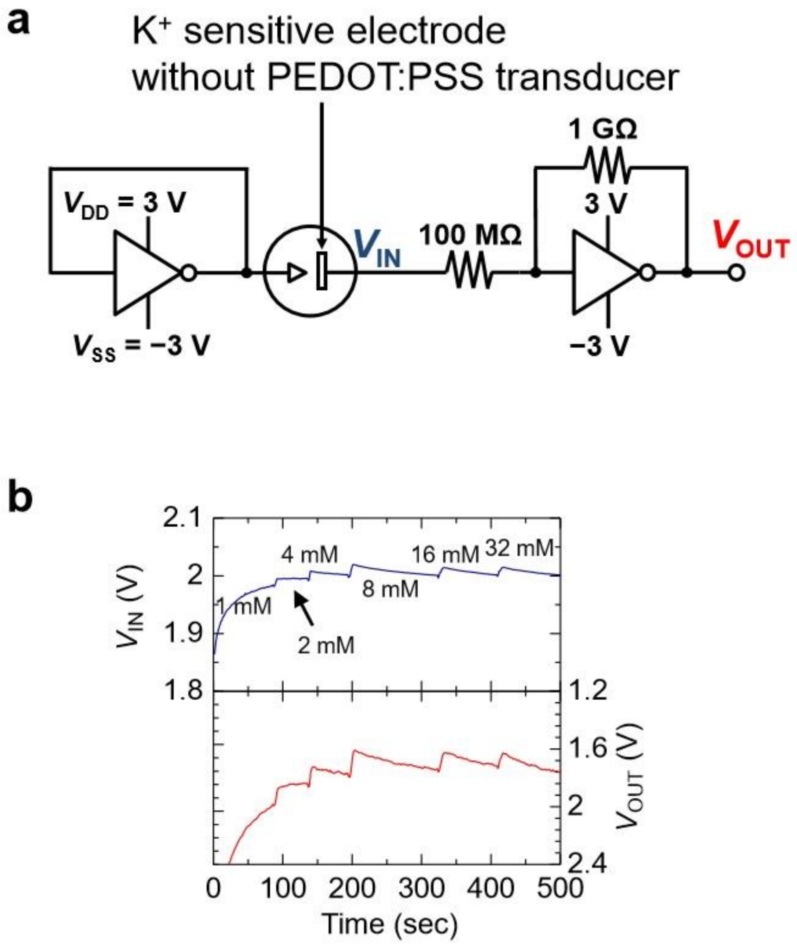

Figure S7. Amplification of small signals from a K^+ sensor without ion-electron transducer. (a) Circuit diagram of the amplifier. Supply voltage ($V_{DD} = -V_{SS}$) and control voltage (V_C) of the reference inverter and the amplification inverter were set to 3 V and 2 V, respectively. (b) Input voltage (V_{IN}) and output (V_{OUT}) from the amplifier system. The voltages were not stable owing to the current flow through the ion-sensitive electrode (ISE).
